# Knowledge of physicians regarding the management of Type two Diabetes in a primary care setting: the impact of online continuous medical education

**DOI:** 10.1186/s12909-020-02212-3

**Published:** 2020-10-20

**Authors:** Zahra Emami, Azam Kouhkan, Alireza Khajavi, Mohammad E. Khamseh

**Affiliations:** 1 Endocrine Research Center, Institute of Endocrinology and Metabolism, Iran University of Medical Sciences (IUMS), Tehran, Iran, No. 10, Firoozeh St, Vali-asr Ave, Vali-asr Sq, Tehran, Iran; 2 Reproductive Epidemiology Research Center, Royan Institute for Reproductive Biomedicine, ACECR, Tehran, Iran; 3 Department of Diabetes, Obesity and Metabolism, Cell Science Research Center, Royan Institute for Stem Cell Biology and Technology, ACECR, Tehran, Iran; 4 Student Research Committee, Faculty of Paramedical Sciences, Shahid Beheshti University of Medical Sciences, Tehran, Iran

**Keywords:** CME; continuing medical education, Internet, Primary care, Physicians, Type 2 diabetes

## Abstract

**Background:**

To explore the impact of an online continuing medical education (CME) program on physicians’ knowledge about the management of type two diabetes.

**Methods:**

An online CME program was designed and uploaded in the CME platform, Department of Education, Ministry of health, Iran. A 28-item questionnaire was used for the assessment. In the beginning, a case scenario was introduced. Then, participants were asked to follow and answer to a pretest assessment. Details of the educational content were provided afterward. Finally, the participants took part in the same post-test exam 4 weeks later. The Wilcoxon matched-pairs signed-ranks test was used to compare the measurements. In addition, the Mann-Whitney test was applied to compare knowledge indices between the general practitioners (GPs) and internists.

**Results:**

Five hundred twenty-six primary care physicians participated in this study. There was a significant positive effect regarding diagnosis confirmation (10.3% difference, *P* = 0.0001). Moreover, a smaller effect was observed in relation to the importance of glycosylated hemoglobin (HbA1c) at diagnosis (5.2% difference, *P* = 0.0006). The effect was positive in relation to the self-reported HbA1c testing frequency: more than 90% of the participants answered correctly in the post-test exam (7.6% difference, *P* = 0.0001). Considering improved knowledge in the treatment of diabetes, there was a very significant difference in response to questions targeting advice on a healthy diet, and physical activity; 27.7% (*P* = 0.000), and 18.7% (P = 0.000), respectively. In addition, the program had a positive impact on various aspects of treatment with oral glucose-lowering drugs (OGLDs). Moreover, the intervention difference was 25, and 34.4% for the questions targeting the appropriate type of insulin, and insulin initiation regimen after OGLD failure. Subgroup analyses revealed that the intervention increased the rate of correct responses among the GPs in various domains of knowledge in diagnosis and treatment. The initial differences between the GPs and internists no longer remained significant after the intervention.

**Conclusion:**

Knowledge of Iranian primary health care professionals in diabetes management has significant shortcomings. This is concerning because they are at the front line of patient care. We demonstrate the effectiveness of online CME on improving GPs knowledge in the management of type 2 diabetes.

## Background

Diabetes, one of the most common endocrine disorders, is a leading cause of death worldwide [1]. The prevalence of diabetes is estimated to increase from 425 to 629 million by 2045 [2]. More than 9% of diabetic patients are from the Middle East and North Africa [3]. In Iran, 11.5% of the adult population has diabetes. Approximately 50% of deaths related to diabetes occur in patients under the age of 60 years [4]. Hence, timely diagnosis and good control of diabetes may prevent or delay the development of long-term adverse outcomes [5].

Primary healthcare professionals are frontline care providers in the management of diabetes and its complications [6–8]. The complexity and chronicity of diabetes are challenging for healthcare professionals; thus improving and updating physician^’^s knowledge and performance through education is essential [9]. The main objectives of diabetes management are to control risk factors, achieve desired glycemic goals, and prevent diabetes-related complications in order to improve patients’ quality of life [9]. In this context, continuing medical education (CME) is defined as any activity applied to maintain, develop, and improve the knowledge, skills, and professional performance of physicians [10, 11].

The disadvantages of traditional CME include being teacher-oriented, having an in-person-based approach, high cost, low educational quality, time constraints, widespread geographical distribution, and time away from work [12]. The emerging concept of CME concurrent with the increasing growth of information technology and electronic devices have led specialists to consider electronic education as a substitute or supplementary method in the field of education in recent years [13, 14].

Online CME as a web-based technology has been validated as an effective modality to make changes in the knowledge and behavior of physicians and to improve their confidence [15–17]. It is searchable, accessible, and on-demand. It is cost-effective and a strong source of CME for a large audience in their busy lives [18]. Today, the use of online CME is growing [19]. Numerous studies have found online CME to be a useful tool in palliative care training for healthcare professionals [20, 21]. Recently, a systematic review reported the positive impacts of online CME regarding the knowledge and satisfaction of general practitioners (GPs); however, the best delivery method is yet to be determined [22].

Several investigations have evaluated the beneficial effects of online CME programs on the medical knowledge, practice, and behavior of healthcare providers [22, 23]. In recent years, online CME activities have focused on serious illnesses such as diabetes in different groups of healthcare providers including GPs [24], nurses [25], and internal medicine physicians [26]. Most studies have indicated that online CME interventions improve participants’ diabetes knowledge and management skills in the areas of nutrition [27], monitoring metabolic markers [26], and managing dyslipidemia [28], as well as the clinical knowledge and confidence of physicians in using new medications, including GLP1 receptor agonists [29]. However, the influence of online CME programs based on American Diabetes Association (ADA)/ the European Association for the Study of Diabetes (EASD) guidelines [30] on the knowledge of healthcare providers, especially GPs and internists, is not clearly understood Previously, we showed that 40% of Iranian primary healthcare professionals took part in CME-accredited programs in diabetes, the majority being from large cities [31]. Hence, online CME could potentially provide an opportunity for equality in education. Currently, certified academic centers are responsible for delivering CME programs in Iran. The impact of the programs should be evaluated in order to improve their content and the methods of delivery. Hence, the aim of this study was to explore the impact of an online CME program on physicians’ knowledge in the diagnosis and treatment of type 2 diabetes in the primary care setting.

## Methods

This cross-sectional study was conducted on physicians involved in the management of diabetes in a primary healthcare setting. The Institute of Endocrinology and Metabolism is one of the certified academic centers eligible to run online CME programs in Iran. All online CME programs are uploaded in a platform provided by the Deputy of Education, Ministry of Health, available at http://ircmelms.ir. Healthcare professionals from all over the country have access to this platform and can participate in the programs according to their educational needs. The current online CME program was uploaded on the platform and active from November 21, 2017 until November 22, 2018.

The sample size was calculated to be 531, using the formula $n=\frac{{z_{\frac{\alpha }{2}}}^2{\sigma}^2}{d^2}$, a confidence level of 0.05, absolute error value of *d* = 1.05, and standard error for knowledge of 12.35 [32]. Excluding 5 participants who did not answer to the post test questionnaire, data from 526 participants who completed both pre-test and post-test questionnaires were analyzed.

**Fig. 1:**
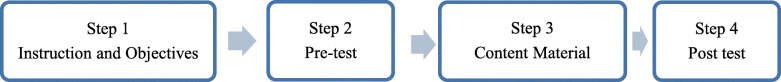
Online CME process steps

The program was designed in four steps. Initially, the participants were provided with the instructions and the main objectives. In the second step, they were asked to follow and answer to the pre-test questions. Then, the details of the educational content, including a video presentation, PowerPoint slides, and useful links to the ADA/EASD guidelines [30] were provided to them. The video presentation contained the most relevant content and a step-wise approach to the diagnosis and treatment of type 2 diabetes based on the latest ADA/EASD guideline. The presenting speaker was an endocrinologist with good expertise in diabetes. The PowerPoint slide presentation included details of the latest evidence in the management of diabetes. Finally, the participants were given an opportunity to respond to the post-test exam 4-weeks later. They were not allowed access to the pre-test or content material at the time of the final assessment. The process steps are illustrated in Fig. 1.

A 28-item questionnaire consisting of three sections was used to assess the knowledge of the participants. The first section included questions on demographic and professional characteristics of the participants. The second section consisted of six multiple choice questions related to diagnosis (Q7, Q8, and Q9) and glycemic control (Q20, Q21, and Q22). The third section focused on the treatment of diabetes and consisted of 16 multiple choice questions related to lifestyle modification (Q10, Q11, Q12), use of oral glucose-lowering drugs (OGLDs) (Q13, Q14, Q15, Q16, Q17, Q18, Q19, Q23), and insulin therapy (Q24, Q25, Q26, Q27, Q28). The following case scenario was presented at the beginning:

*“A 42-year-old man is referred to you because of a high blood sugar level detected during a screening program. He denies any symptoms related to hyperglycemia. His father had type 2 diabetes and died at age 68. There is no history of any medical problem in the past. Drug history is negative.*

*In physical examination, height is 170 cm, body weight is 90 kg, and BMI=30 kg/m2.*

*Fasting plasma sugar is 212 mg/dl.*

*What would be your next step to confirm the diagnosis of diabetes in this patient?”*

To assess the questionnaire’s reliability, a pilot study was performed on 100 participants. Cronbach’s alpha was equal to 87%, reflecting a good level of reliability. In order to evaluate the validity of the questionnaire, six endocrinologists evaluated the questions for necessity, relevance, clarity, and simplicity. Then, the content validity index (CVI) and content validity ratio (CVR) were calculated. The overall CVI, relevance CVI, clarity CVI, and simplicity CVI were 0.812, 0.861, 0.906, and 0.824, respectively. The critical point of 0.75 was selected for the CVR [33]. All individual questions obtained CVRs ranging between 0.783 and 1.

### Statistical analysis

All data was collected in an Excel file, and STATA version 10 was used for analysis of the data. Continuous variables were reported as mean (SD) and discrete ones as numbers (proportions). The equality of matched-pair (before/after) observations was assessed using the Wilcoxon matched-pairs signed-ranks test. The Mann-Whitney test was used to compare the knowledge indices regarding diagnosis and treatment scores between GPs and internists. The Pearson chi-squared and Fisher tests were used to compare GPs and internists, in either the before or after measurement times.

### Ethical approval

Ethical approval was received from the Iran University of Medical Sciences ethics committee. (IR, IUMS.REC.1392.24217).

## Results

Overall, 526 participants completed both the pre-test and post-test assessments. Table 1 shows the characteristics of the study participants. Fifty-one percent of the physicians had previously taken part in accredited CME activities; 57% were working in a large city, and 62% were employed in public healthcare services.

**Table 1:** Baseline characteristics of the study participant (*n*: 526)

Variable		%
Past CME credit (yes)	Accredited CME (*n* = 265)	51
	Non-accredited CME (*n* = 261)	49
Number of visited patients per month	< 10 (*n* = 408)	78
	> 10 (*n* = 118)	22
Workplace	City (*n* = 297)	57
	Town/ village (*n* = 229)	43
Time since graduation (yrs.)	< 10 (*n* = 190)	36
	> 10 (*n* = 336)	64
Clinical practice(yrs.)	< 10 (*n* = 287)	54
	> 10 (*n* = 239)	46
Healthcare facility	Public (*n* = 325)	62
	Private (*n* = 201)	38

Table 2 indicates the effect of the intervention on the knowledge of the participants regarding diagnostic approach and glycemic control. There was a significant positive effect regarding Q7 (diagnosis confirmation, 10.3% difference, *p* = 0.000). A smaller but significant effect was observed in relation to the importance of glycosylated hemoglobin (HbA1c) at diagnosis (Q8, *p* = 0.0006). However, there was no significant difference in regard to the importance of renal and thyroid function assessment or the lipid profile measurement (Q9). The participants’ knowledge of glycemic control was explored. More than 80% of the participants answered correctly to the question targeting the goal of HbA1c after treatment initiation (Q21). The effect of CME was considerable in relation to the HbA1c testing frequency; more than 90% of the participants answered correctly in the post-test. The intervention had a negative impact on the most appropriate test for assessing glycemic control (Table 2).

**Table 2:** Effect of the on-line CME program on participants’ knowledge in diagnosis and glycemic control (*n* = 526)

Question	Diagnostic approach			*P*-value	Glycemic Control			*P*-value
	Pre-test Correct answer (%)	Post- test Correct answer (%)	Diff (%)		Pre-test Correct answer (%)	Post-test Correct answer(%)	Diff (%)	
Q7: Diagnosis confirmation	57.4	67.7	10.3	0.000				
Q8: Importance of HbA1c at diagnosis	52.9	58.1	5.2	0.0006				
Q9: Renal and thyroid function assessment	87.6	86.3	−1.3	0.3				
Q20: FBS and HbA1c in follow up					77.9	69.9	−8	0.0003
Q21: Goal of HbA1c					80.9	80.6	−0.3	0.846
Q22: HbA1c test- frequency					75.8	93.4	17.6	0.0001

Wilcoxon matched pairs test was used to compare before/after assessments

The effect of the intervention on knowledge of physicians in relation to the treatment of diabetes is shown in Table 3. More than 90% of the participants recommended lifestyle modification from the onset of diabetes. There was a very significant difference in responses to questions Q11 and Q12 which targeted lifestyle change recommendations (27.7% (*p* = 0.000) and 18.7% (*p* = 0.000) intervention differences, respectively). With regard to therapy with OGLDs, there was a significant difference between the pre- and post-intervention responses to Q13 (“Do you recommend medical treatment concurrently with changes in lifestyle modification for diabetes?”), Q15 (“Do you recommend combination therapy at diagnosis for patients with relatively high HbA1c levels?”), and Q16 (“At what HbA1c value should we combine antihyperglycemic drugs?). Moreover, the effect of the intervention on starting metformin as the first OGLD (Q17, intervention difference: 16.6%, *p* = 0.000), the optimal dose of sulfonylurea (Q19, intervention difference: 24.4%, *p* = 0.000), and the highest effective dose for metformin (Q23) were significant. Conversely, there was no difference in responses to Q14, which targeted the initiation of OGLDs concurrent with lifestyle recommendations, or Q18, which asked about metformin dose at initiation. Considering insulin therapy, there was a significant difference between pre-intervention and post-intervention responses to questions 24 to 28 regarding insulin initiation, type of insulin, and dose titration. The intervention differences were 25 and 34.4% for questions asking the appropriate type of insulin and insulin initiation regimen after OGLD failure, respectively (Table 3).

**Table 3:** Effect of the on-line CME program on participants’ knowledge in treatment of diabetes (*n*: 526)

Life style Modification	OGLDs Treatment								Insulin Therapy			
Question	Pre-test	Post-test	Diff (%)	*P*-value	Pre-test	Post-test	Diff (%)	*P*-value	Pre-test	Post-test	Diff (%)	*P*-value
Q10: Lifestyle modification recommendation	90.8	93.1	2.3	0.1								
Q11: Advice on healthy diet	46.6	74.3	27.7	0.000								
Q12: Physical activity advice	60.0	78.7	18.7	0.000								
Q13: Medical treatment+ life style change					66.1	82.1	15.9	0.000				
Q14: Concurrent medical therapy					68.8	68.8	0	1.000				
Q15: Combination therapy					46.7	71.1	24.3	0.000				
Q16: HbA1c level to recommend combination therapy					54.1	85.7	31.6	0.000				
Q17: First choice OGLD					71.8	88.4	16.6	0.000				
Q18: Metformin dose at initiation					59.6	63.8	4.2	0.0518				
Q19: Optimal dose of sulfonylurea					62.7	89.1	24.4	0.000				
Q23: Metformin dose titration					45.2	50.9	5.7	0.005				
Q24: Adding basal insulin									61.7	86.5	24.8	0.000
Q25: Insulin regimen									19.9	54.3	34.4	0.000
Q26: Basal insulin dose titration based on FBS									22.2	38.3	16.1	0.000
Q27: Indication for up-titration of basal insulin									58.5	68.4	9.9	0.000
Q28:Indication for adding prandial insulin									63.5	76.5	13	0.000

Wilcoxon matched pairs test was used to compare before/after assessments

The means of knowledge indices in diagnosis and treatment scores before and after the educational intervention are presented in Table 4. There was a significant difference in the scores after the intervention (*p*-value for both = 0.0001). The impact of the intervention was more pronounced on the treatment of participants.

**Table 4:** Knowledge indices in diagnosis and treatment of diabetes before and after the intervention

	*Before	*After	*P*-value
Diagnosis	4(3–5)	5(4–5)	0.0001
Treatment	9(7–13)	12(11–13)	0.0001

Interquartile range)* median)

Mann-Whitney test was used

Table 5 indicates the difference in knowledge of general physicians and internists before and after the intervention. Regarding the diagnostic approach, a significantly higher proportion of internists gave the correct response to Q8 before and after the intervention (*p* = 0.025). However, there were no significant differences between the two groups in relation to the importance of diagnosis confirmation or the importance of renal/thyroid function assessment and lipid profile measurement. With regard to knowledge on glycemic control, a significantly higher proportion of internists gave the correct response to the question about HbA1c testing frequency before the intervention (*p* = 0.036). The difference was no longer significant after the intervention. Furthermore, no difference was observed between the groups in relation to the questions targeting the goal of HbA1c or the most appropriate test for assessing glycemic control (Table 5).

**Table 5:** General physicians’ and internists’ knowledge in diagnosis of diabetes before and after intervention

Diagnostic approach						Glycemic Control	
Question		GPs	Internist	*P-*value	GPs	Internist	*P*-value
Q7	Pre-test	58.2	47.0	0.202			
	Post-test	68.4	55.8	0.13			
Q8	Pre-test	50.7	70.5	0.025			
	Post-test	56.8	76.4	0.025			
Q9	Pre-test	86.9	97.0	0.105			
	Post-test	85.7	94.1	0.206			
Q20	Pre-test				78.4	70.5	0.288
	Post-test				69.6	73.5	0.634
Q21	Pre-test				80.6	85.2	0.505
	Post-test				80.0	88.2	0.368
Q22	Pre-test				74.7	91.1	0.036
	Post-test				82.6	94.1	0.096

Chi-squared (or Fisher exact test) was used, depending on the percentages inside the tables to be large or small, respectively

Next, the effects of the online CME program on the treatment of GPs and internists were compared (Table 6). A significantly higher proportion of internists gave the correct response to Q12 targeting lifestyle modification before the intervention (*p* = 0.043). However, the impact of the intervention on GPs was substantial. Hence, there was no significant difference between the two groups in their responses to this question after the intervention.

**Table 6:** General physicians’ and internists’ knowledge in treatment of diabetes before and after educational intervention

Life style Modification					OGLDs Treatment			Insulin therapy		
Question		GPs	Internist	*p*-value	GPs	Internist	*p*-value	GPs	Internist	*p*-value
Q10	Pre-test	90.2	100	0.061						
	Post-test	92.8	97.0	0.5						
Q11	Pre-test	50.5	38.2	0.166						
	Post-test	73.9	79.4	0.479						
Q12	Pre-test	58.8	76.4	0.043						
	Post-test	78.2	85.2	0.393						
Q13	Pre-test				64.9	82.3	0.038			
	Post-test				82.4	76.4	0.376			
Q14	Pre-test				67.8	82.3	0.077			
	Post-test				68.0	79.4	0.166			
Q15	Pre-test				47.0	44.1	0.741			
	Post-test				69.8	88.2	0.02			
Q16	Pre-test				53.7	61.7	0.365			
	Post-test				85.7	85.2	1.000			
Q17	Pre-test				70.3	85.2	0.078			
	Post-test				87.9	94.1	0.0408			
Q18	Pre-test				59.4	64.7	0.547			
	Post-test				62.9	76.4	0.112			
Q19	Pre-test				63.5	50	0.114			
	Post-test				89.2	88.2	0.778			
Q23	Pre-test				46.2	29.4	0.057			
	Post-test				52.1	35.2	0.057			
Q24	Pre-test							60.2	85.2	0.003
	Post-test							86.3	88.2	1.000
Q25	Pre-test							18.9	35.2	0.021
	Post-test							53.2	70.6	0.048
Q26	Pre-test							21.5	32.3	0.145
	Post-test							37.8	47.0	0.288
Q27	Pre-test							59.0	50	0.3
	Post-test							68.4	67.6	0.924
Q28	Pre-test							60.2	82.3	0.010
	Post-test							75.4	90.9	0.054

Chi-squared (or Fisher exact test) was used, depending on the percentages inside the tables to be large or small, respectively

Table 6 summarizes the changes effected by the educational intervention in the knowledge of diabetes treatment among the participants. With respect to OGLD therapy, a significantly higher proportion of internists gave the correct response to Q13 (“Recommending medical treatment concurrently with changes in lifestyle modification for diabetes”) before the intervention (*p* = 0.043). However, there was about 18% increase in the rate of correct responses to this question among GPs. Therefore, the difference disappeared after the intervention. There was also a significant difference between the groups in response to Q15 after the intervention (*p* = 0.02). This question targeted the importance of combination therapy at diagnosis in patients with high HbA1c levels. There was 40% increase in the rate of correct responses among internists after the intervention. A significantly higher proportion of internists, compared to GPs, gave the correct answer to Q24 (type of insulin at initiation) before the intervention (*p* = 0.003); however, the intervention increased the rate of correct responses among GPs by 26%. Therefore, the difference was no longer significant after the intervention. Moreover, 35% of GPs and internists gave the correct response to Q25 (“basal insulin therapy at insulin initiation”) after the intervention. Nevertheless, the rate of correct response was greater among the internists: 70.6% vs. 53.2% (Table 6).

## Discussion

Improvement in the quality of diabetes care is a major challenge for healthcare systems all over the world [34]. In this context, the role of primary health care providers can not be overemphasized. Care for chronic conditions such as diabetes mellitus needs patient engagement and empowerment as well as improvement in the knowledge and skills of general physicians and internists in both the short and longterm [34]. In the current century, the virtual world provides online CME to keep graduate clinicians’ knowledge and practice up-to-date, which consequently leads to better patient care processes and outcomes [24]. The present study assessed the impact of online CME on the knowledge of physicians in the management of type 2 diabetes in Iran.

The results showed that approximately 57% of physicians were employed in large cities and mostly in public healthcare services. Fifty-one percent of them had previously taken part in a traditional CME activity. The intervention was effective in increasing the knowledge of the participants regarding the diagnosis of diabetes and glycemic control. Most of the participants gave correct answers to the questions targeting the goal of HbA1c (80%) and HbA1c testing frequency (90%) after the post-test. Currently, good glycemic control is a cornerstone of diabetes care and HbA1c (a valuable test for clinical decision making) is the key biomarker of long-term glycemic control [35]. The positive impact of the intervention on the participants’ knowledge of good glycemic control and HbA1c testing may improve the quality of diabetes care. Crenshaw et al. (2010) found the impact of web-based interventions and CME programs was related to the level of participation; because doctors of the patients with the worst A1c control were too busy to participate in these educating progams [26]. Gallardo-Rincon et al. (2017) in the CASAUD Model described the linkages between health care, diabetes patient health knowledge, diabetes self-management activities, and some diabetes biomarkers. This study concluded online CME is a feasible strategy for improvement in quality of health care [36].

The impact of the online CME program on physicians’ knowledge in the management of diabetes through lifestyle modification, use of OGLDs, and insulin therapy was evaluated. More than 90% of the participants recommended lifestyle modification from the onset of diabetes. Advice on a healthy diet and physical activity improved after the post-test. There was a significant improvement in the appropriate use of OGLDs and a stepwise approach to insulin therapy recommendations after the intervention. Proper OGLDs therapy and early insulin intensification with patient self-management education are essential for better outcomes and for reducing the risk of long-term complications of diabetes. Physician-related barriers may be overcome by providing physicians with education and training. Moreover, online CME programs keep physicians updated about current diabetes guidelines [37]. Similar to our finding, Hicks & Murano (2017) concluded lifestyle-focused online CME programs improved knowledge of physicians [27]. In addition, recent studies emphasized the success of online video-based CME on improving knowledge of primary care physicians related to diabetes management and insulin therapy [38, 39].

Numerous systematic reviews and meta-analyses have assessed the effectiveness of CME [40–44] and online CME [20, 22] interventions on physicians’ knowledge, skills, and professional performance. In a sysnthesis of 39 systematic reviews, Cervero et al. (2015) concluded that CME activity leads to more positive outcomes if it has several characteristics, including being more interactive, using more methods, involving multiple exposures, lasting longer, and being focused on outcomes. However, few studies have examined the effects of online CME on the knowledge and practice of professional health practitioners in the management of type 2 diabetes [45]. Beaser et al. (2013) recommended a new approach for diabetes-focused CME and emphasized performance improvement CME [46]. A recent publication reported that a one-hour online CME course can assist practicing physicians in addressing patient nutrition and lifestyle concerns related to type 2 diabetes [27]. Similar to the current research, this publication reported the positive impact of a single online diabetes-focused CME on physicians’ knowledge; however, the sample size in the reported study was low (especially concerning GPs and medical internists) and covered various areas of practice specialties and nutrition-focused CME on Type 2 diabetes [27].

The present study also compared the effect of online CME on GPs and internists. Overall, internists had a better knowledge of diabetes management than GPs. Subgroup analyses revealed that the intervention increased the rate of correct responses among GPs in various domains of knowledge. Hence, the differences no longer remained significant after the intervention. In other words, taking part in the program was more effective for the GPs as the frontline physicians to tackle diabetes management. A cross-sectional study revealed a lack of knowledge, attitude, and practice among primary healthcare physicians, especially family physicians working in rural areas, regarding the management of diabetes [47]. Katulanda et al. [9] suggested the knowledge and practices related to diabetes of GPs in developing countries needs to be updated regarding diagnostic approach, monitoring glycemic control, and setting appropriate glycemic targets by sustained medical education and training programs. A previous survey indicated that the knowledge and practice of Iranian GPs in the management of type 2 diabetes were insufficient, and traditional CME programs were not effective in changing their clinical practice [31]. Niroomand et al. [48] evaluated the knowledge, attitude, and practice of Iranian internists regarding diabetes mellitus and concluded that age and time since graduation were inversely correlated with internists’ knowledge and practice, except for physicians who worked in teaching hospitals. Lim et al. (2020) reported significant improvements in diabetes-related knowledge, skills, and clinical practice among general physicians and nurses through a six-month training program called the Steno REACH Certificate Course in Clinical Diabetes Care (SRCC) [49].

In the current study, the impact of online CME on physicians’ knowledge regarding diabetes management was examined. One of the strengths of the study is that physicians from small cities and villages had the opportunity to participate. Moreover, this study showed the effectiveness of online CME intervention on GPs’ knowledge in diabetes management. However, the short time period for post-intervention assessment is a limitation of this study. Future research should investigate the impact of online programs in clinical practice and patients’ outcomes over a long-term period.

## Conclusions

In summary, this study demonstrated that knowledge of diabetes among Iranian primary healthcare professionals has significant shortcomings. It further demonstrated the effectiveness of an online CME program in the knowledge of general practitioners regarding the management of type 2 diabetes. In addition, up-to-date online CME is a good option for expanding the number of certified primary healthcare professionals in diabetes management.

## Data Availability

The datasets used and/or analyzed during the current study are available from the corresponding author on reasonable request.

## Abbreviations

ADA/EASD: American Diabetes Association (ADA)/ the European Association for the Study of Diabetes (EASD)
CME: Continuing Medical Education
CVI: Content Validity Index
CVR: Content Validity Ratio
GPs: General Practitioners
HbA1c: Glycosylated Hemoglobin
IDF: International Diabetes Federation
OGLDs: Oral Glucose-Lowering Drugs
SD: Standard Deviation

## References

[1] Shaw JE, Sicree RA, Zimmet PZ (2010). Global estimates of the prevalence of diabetes for 2010 and 2030. Diabetes Res Clin Pract.

[2] International Diabetes Federation. IDF Diabetes Atlas, 8th ed. Brussels, Belgium:] International Diabetes Federation, 2017. http://www.diabetesatlas.org; last accessed on January 23, 2019.

[3] Ogurtsova K, da Rocha Fernandes JD, Huang Y, Linnenkamp U, Guariguata L, Cho NH (2017). IDF diabetes atlas: global estimates for the prevalence of diabetes for 2015 and 2040. Diabetes Res Clin Pract.

[4] Esteghamati A, Etemad K, Koohpayehzadeh J, Abbasi M, Meysamie A, Noshad S (2014). Trends in the prevalence of diabetes and impaired fasting glucose in association with obesity in Iran: 2005–2011. Diabetes Res Clin Pract.

[5] Nguyen Q, Nguyen L, Felicetta J (2008). Evaluation and management of diabetes mellitus. Am Health Drug Benefits.

[6] Goyder EC, McNally PG, Drucquer M, Spiers N, Botha JL (1998). Shifting of care for diabetes from secondary to primary care, 1990–5: Review of general practices. BMJ..

[7] Khunti K, Ganguli S (2000). Who looks after people with diabetes: primary or secondary care?. J R Soc Med.

[8] Drass J, Kell S, Osborn M, Bausell B, Corcoran J, Moskowitz A (1998). Diabetes care for medicare beneficiaries, attitude and behaviors of primary care physcians. Diabetes Care.

[9] Katulanda P, Constantine GR, Weerakkody MI, Perera YS, Jayawardena MG, Wijegoonawardena P (2011). Can we bridge the gap? Knowledge and practices related to diabetes mellitus among general practitioners in a developing country: a cross sectional study. Asia Pac Fam Med.

[10] Lewis C (1998). Continuing medical education: past, present, future. West J Med.

[11] Shumway JM, Harden RM (2003). AMEE guide no. 25: the assessment of learning outcomes for the competent and reflective physician. Med Teach.

[12] Stricker D, Weibel D, Wissmath B (2011). Efficient learning using a virtual learning environment in a university class. Comput Educ.

[13] Smits PBA, Graaf LD, Radon K, AGD B, Bos NR, FJH D (2012). Case-based e-learning to improve the attitude of medical students towards occupational health, a randomised controlled trial. Occup Environ Med.

[14] Bonnel W (2008). Improving feedback to students in online courses. Nurs Educ Perspect.

[15] Curran V, Lockyer J, Sargeant J, Fleet L (2006). Evaluation of learning outcomes in web-based continuing medical education. Acad Med.

[16] Vollmar HC, Schurer-Maly CC, Frahne J, Lelgemann M, Butzlaff M (2006). An e-learning platform for guideline implementation-evidence- and case-based knowledge translation via the internet. Methods Inf Med.

[17] Cook DA, Levinson AJ, Garside S, Dupras DM, Erwin PJ, Montori VM (2008). Internet-based learning in the health professions. JAMA..

[18] Casebeer L, Engler S, Bennett N, Irvine M, Sulkes D, DesLauriers M (2008). A controlled trial of the effectiveness of internet continuing medical education. BMC Med.

[19] Harris JM, Sklar BM, Amend RW, Novalis-Marine C (2010). The growth, characteristics, and future of online CME. J Contin Educ Heal Prof.

[20] Weston CM, Sciamanna CN, Nash DB (2008). Evaluating online continuing medical education seminars: evidence for improving clinical practices. Am J Med Qual.

[21] Pelayo M, Cebrián D, Areosa A, Agra Y, Izquierdo JV, Buendía F (2011). Effects of online palliative care training on knowledge, attitude and satisfaction of primary care physicians. BMC Fam Pract.

[22] Thepwongsa I, Kirby CN, Schattner P, Piterman L (2014). Online continuing medical education (CME) for GPs: does it work? A systematic review. Aust Fam Physician.

[23] Curran VR, Fleet L (2005). A review of evaluation outcomes of web-based continuing medical education. Med Educ.

[24] Paul CL, Piterman L, Shaw JE, Kirby C, Forshaw KL, Robinson J (2017). Poor uptake of an online intervention in a cluster randomised controlled trial of online diabetes education for rural general practitioners. Trials..

[25] Moattari M, Moosavinasab E, Dabbaghmanesh MH, ZarifSanaiey N (2014). Validating a web-based diabetes education program in continuing nursing education: knowledge and competency change and user perceptions on usability and quality. J Diab Metab Disord.

[26] Crenshaw K, Curry W, Salanitro AH, Safford MM, Houston TK, Allison JJ (2010). Is physician engagement with web-based CME associated with patients' baseline hemoglobin A1c levels? The rural diabetes online care study. Acad Med.

[27] Hicks KK, Murano PS (2017). Online nutrition and T2DM continuing medical education course launched on state-level medical association. Adv Med Educ Pract.

[28] Spyropoulos J, Healy C. Improving Management of Diabetic Dyslipidemia-Effect of Online CME. Diabetes. 2018;67(Suppl 1):2215-PUB.

[29] Larkin A, Lacouture M, Le A. Online CME Improves Clinical Knowledge and Confidence Related to Use of GLP-1 Receptor Agonists in Combination with Basal Insulin. Diabetes 2019; 68(Suppl 1):2258-PUB.

[30] American Diabetes Association (2016). Standards of medical Care in Diabetes—2017: summary of revisions. Diabetes Care.

[31] Aghili R, Malek M, Baradaran HR, Peyvandi AA, Ebrahim Valojerdi A, Khamseh ME (2015). General practitioners’ knowledge and clinical practice in management of people with type 2 diabetes in Iran; the impact of continuous medical education programs. Arch Iran Med.

[32] Niroomand M, Ghasemi SN, Karimi-Sari H, Kazempour-Ardebili S, Amiri P, Khosravi MH (2016). Diabetes knowledge, attitude and practice (KAP) study among Iranian in-patients with type-2 diabetes: a cross-sectional study. Diab Metab Syndr.

[33] Lawshe CH (1975). A quantitative approach to content validity. Pers Psychol.

[34] Al-Alawi K, Al Mandhari A, Johansson H (2019). Care providers’ perceptions towards challenges and opportunities for service improvement at diabetes management clinics in public primary health care in Muscat, Oman: a qualitative study. BMC Health Serv Res.

[35] Krhač M, Lovrenčić MV (2019). Update on biomarkers of glycemic control. World J Diabetes.

[36] Gallardo-Rincón H, Saucedo-Martínez R, Mujica-Rosales R, Lee EM, Israel A, Torres-Beltran B (2017). Online continuing medical education as a key link for successful noncommunicable disease self-management: the CASALUD™ model. Diab Metab Syndr Obes.

[37] Kalra S, Deb P, Gangopadhyay KK, Gupta S, Ahluwalia A (2019). Capacity and confidence building for general practitioners on optimum insulin use. J Family Med Prim Care.

[38] Larkin A, Dropkin J, Le A. Follow-on basal insulin-can online CME improve clinical knowledge and ability to use effectively? Diabetes. 2018;67(Supplement 1). 10.2337/db18-1005-P.

[39] Larkin A, Healy CS, Le A. Evidence-based basal insulin use in T2D: extensive knowledge and confidence increases with CME. Diabetes. 2019;68(Supplement 1). 10.2337/db19-2257-PUB.

[40] Davis D, Galbraith R (2009). American College of Chest Physicians Health and Science Policy Committee. Continuing medical education effect on practice performance: effectiveness of continuing medical education: American college of chest physicians evidence-based educational guidelines. Chest..

[41] Forsetlund L, Bjørndal A, Rashidian A, Jamtvedt G, O'Brien MA, Wolf F (2009). Continuing education meetings and workshops: effects on professional practice and health care outcomes. Cochrane Database Syst Rev.

[42] Mansouri M, Lockyer J (2007). A meta-analysis of continuing medical education effectiveness. J Contin Educ Heal Prof.

[43] Marinopoulos SS, Dorman T, Ratanawongsa N, Wilson LM, Ashar BH, Magaziner JL (2007). Effectiveness of continuing medical education. Evid Rep Technol Assess.

[44] Mazmanian PE, Davis DA, Galbraith R (2009). American College of Chest Physicians Health and Science Policy Committee. Continuing medical education effect on clinical outcomes: effectiveness of continuing medical education: American college of chest physicians evidence-based educational guidelines. Chest..

[45] Cervero RM, Gaines JK (2015). The impact of CME on physician performance and patient health outcomes: an updated synthesis of systematic reviews. J Contin Educ Heal Prof.

[46] Beaser RS, Brown JA (2013). Preventive intervention in diabetes: a new model for continuing medical education. Am J Prev Med.

[47] Abu Kahf MMM, Abbas Ayad KM, Ahmed Gabr M, Mohamed EW, Shehata S (2019). Knowledge, Attitudes and Practices of Primary Health Care Physicians Regarding Diabetes Management: A Cross Sectional Study in Gharbia Governorate, Egypt. Asian J Med Health.

[48] Niroomand M, Ghasemi SN, Karimi-sari HR, Khosravi MH (2017). Knowledge, attitude, and practice of Iranian internists regarding diabetes: a cross sectional study. Diabetes Metab J.

[49] Lim SC, Mustapha FI, Aagaard-Hansen J, Calopietro M, Aris T, Bjerre-Christensen U (2020). Impact of continuing medical education for primary healthcare providers in Malaysia on diabetes knowledge, attitudes, skills and clinical practices. Med Educ Online.

